# Preparation and Characterization of Color Photocurable Resins for Full-Color Material Jetting Additive Manufacturing

**DOI:** 10.3390/polym12030650

**Published:** 2020-03-12

**Authors:** Yih-Lin Cheng, Kuan-Chi Huang

**Affiliations:** 1 Department of Mechanical Engineering, National Taiwan University of Science and Technology, Taipei 106, Taiwan; M10403215@mail.ntust.edu.tw; 2 High Speed 3D Printing Research Center, National Taiwan University of Science and Technology, Taipei 106, Taiwan

**Keywords:** material jetting, full-color printing, color photocurable resin, color appearance

## Abstract

Material jetting (MJ)-type 3D printers have been considered as one of the most versatile types of 3D printers, enabling full-color printing and multi-material printing. However, to the best of our knowledge, there are few academic studies on the development of full-color MJ technologies, and the formulation of commercial resins is confidential and proprietary. In this paper, we give an insight into the preparation of photocurable resins in the primary CMYKW (cyan, magenta, yellow, black, and white) colors that are printable with the multiple piezoelectric heads of our homemade MJ full-color 3D printer. The components comprising the resins, such as the photo-initiator, oligomers, monomers, and crosslinkers, were methodically adjusted and characterized to achieve high-performance MJ printable resins. Subsequently, the prepared resins were colored with the CMYKW colors and their ability of high-quality color appearance in full-color printing was demonstrated.

## 1. Introduction

Additive manufacturing (AM), the building of parts layer by layer, is also commonly recognized as 3D printing. It enables manufacturing things that are not feasible through conventional processes like machining or molding, and it is also a faster and material-economical way [1,2,3]. Having been developed for decades, 3D printing now can not only create prototypes, but also directly produce objects in the desired quality, boosting product design progress and performing direct digital manufacturing (DDM) [4,5]. Along with the achieved shape and geometric realization, the color realization of the printed parts has become significantly tempting, especially for people in the fields of art, reproduction, mapping, medicine, education, etc. [6,7,8] There are some commercially available full-color 3D printers in the binder jetting (BJ), sheet lamination, and material jetting (MJ) categories, classified by ISO/ASTM 52900 [9]. 

A BJ-type full-color printer is equipped with inkjet print heads for binders in the four CMYK (cyan, magenta, yellow, and black) primary colors. The binders are selectively jetted over the pre-deposited powder layer, defining the shape and colors. Repetition of the layer-by-layer printing is conducted until the part is completed. The representatives are 3D Systems’ family of ProJet CJP x60 3D printers [10]. The CJP series printers are mainly adopted to generate color models in the prototyping stage during product development and, moreover, simulation models for surgery practice. HP’s Jet Fusion 580 color 3D printer [11] is similar to the BJ process with an additional energy source applied to achieve the fusion of powders. The HP Thermal Inkjet array runs over the full work area, depositing a fusing agent to those powders to build up the model as well as some detailing and coloring agents around the contours, giving the object the accurate desired appearance in a full spectrum. During the run back, the carriage equipped with a fusing energy supplier fuses powders covered with fusing agent. The process repeats, layer-by-layer, until the part is complete. HP’s systems are targeted at production purposes, but applications in prototyping and research and development are also common. The sheet lamination-type full-color printer trims papers to the desired shape with primary color inks jetted on the paper to outline the pattern, and glues the sheets to the adjacent layers. The representative is Mcor ARKePro of Mcor Technologies [12]. Unfortunately, using paper as a base material limits its applications. Moreover, the MJ-type full-color printer directly prints photocurable resins in the CMYKW (cyan, magenta, yellow, black, and white) primary colors to create models. It also can print several distinctive materials—rigid, flexible, or transparent—at once into a single build. The representative is Stratasys J750 and the J8 series of Stratasys [13,14]. J750 has been applied to create multi-material colored human anatomy that mimics bone and tissue, while the J8 series printers are designed to aid designers from early stage prototypes to high fidelity models. MJ-type printers do not require vacuum and sandblasting post-processes to remove the powders, and can print out the model with minimal or no finishing steps [15,16]. The thickness of each layer, the surface finishing, the color appearance, and the printing resolution of MJ color printers are in general superior to the powder-based BJ ones. Hence, MJ printers have been considered as the most reliable and versatile full-color 3D printers.

To the best of our knowledge, we may be the only group dedicated to the development of the MJ full-color 3D printer in the academic field. Our investigation involves mechanical mechanism designs [17,18], automatic print head alignment algorithms [19], and leveling mechanisms [20]. We have also studied 3D color dithering methods for improving color consistency [21]. Acrylate-based photocurable resins were widely used in MJ and vat photopolymerization (VP) 3D printing. Typical acrylate-based photocurable resins are composed of structural oligomers, monomers, photo-initiators, crosslinkers, and additives (such as pigments and filler) [22]. Murphy et al. proposed a resin consisting of both an acrylate and a methacrylate achieving the combined mechanical strength and modulus [23]. Urethane acrylate-based oligomers are commonly used in stereolithography (SLA) patents because their functional groups provide the desired mechanical strength [24]. There is an array of urethane acylate (UA) monomers/oligomers commercially available, which can be further categorized as aliphatic and aromatic-UA, with different degrees of functionality going from one to six [25]. Generally, structural oligomers are too viscous to be directly used in AM processes. Reactive diluents, such as dipropylene glycol diacrylate (DPGDA) and isobornyl acrylate (IBOA), are low viscosity monomers often used to thin the oligomers to form a printable resin [26]. These can be more important because the piezoelectric heads used in MJ printers can only take feeds (either ink or resins) with viscosities from 10~13 cP at the printing temperature of 70~80 °C. Accordingly, an extensively raised amount of monomer should be taken. To facilitate cross-linking for an improved final modulus, multifunctional reactive diluents [27,28] such as trimethyloylpropane triacrylate (TMPTA), pentaerythritol tetraacrylate (PETA), or hyperbranched monomers [29,30,31,32], are often used as crosslinkers. Acylphosphine oxides, such as diphenyl(2,4,6-trimethylbenzoyl) phosphine oxide (TPO), are a class of Type I radical initiators and can be excited to form radical fragments as exposed to higher wavelength light (405 nm) [33], responding to MJ printers or digital light processing (DLP) printers often equipped with lamps at wavelength of 405 nm. The substance added into the resins to be responsible for color is pigments or dyes [34]. The pigment is a powder material, usually in nano-size (~100 nm), that disperses itself in its vehicle without dissolution, while the dye dissolves in its vehicle. Because dyes have been proven to have poor lightfastness, pigments have been frequently employed in ink-printing industries [35].

The formulations of the commercial resins are confidential and proprietary, which hinders the development and utilization of the MJ full-color 3D printer in the academic field. Hence, in this paper, we give an insight into the preparation of MJ-printable resins (or inks) in the primary colors CMYKW. The effects of ink formula adjustments on the physical properties and full-color model quality will be presented and discussed.

## 2. Materials and Methods

### 2.1. Materials and Preparation of Resins

The homemade photocurable resins comprise oligomers (20 wt%) (DBC, New Taipei City, Taiwan; DSM-AGI Co., New Taipei City, Taiwan), a monomer (65~70 wt%) (DBC, New Taipei City, Taiwan), crosslinkers (10~15 wt%) (DBC, New Taipei City, Taiwan; Sartomer Americas, Exton, PA, USA), a photo-initiator (additional 1~5 wt%) (Chembridge, New Taipei City, Taiwan), and additives (additional 0.1~3 wt%) (BASF, Ludwigshafen, Germany; DBC, New Taipei City, Taiwan; ChengFeng Group, Tainan City, Taiwan; ITRI, Hsinchu, Taiwan); the chemicals used for the preparation are listed in Table 1. The proportions of oligomers and crosslinkers indicated in Table 1 were mixed and dissolved into the monomer, and the photo-initiator and additives (such as dispersant and leveling agent) were subsequently added and stirred at 70 °C for 3 h to form an homogeneous resin. The five CMYKW primary color resins for full-color MJ printing were prepared by mixing the preformed photocurable resins with commercially available pigment inks in the CMYKW colors for 6 h and filtered with a 1 μm filter head before placing color resins in the bottles for further testing. 

The effect of concentration of the photo-initiator on volume shrinkage and the degree of conversion were studied first. The selected aliphatic and aromatic polyurethane acrylate (PUA) were compared and formulated by adding monomers and crosslinkers to achieve the appropriate rheological properties (viscosity and surface tension) and promising mechanical properties (ultimate tensile strength and elongation). The best-formulated resin was colored into the five primary colors by adding the pigments. The appearance of the full-color printed samples was demonstrated to show the successful resin preparation.

### 2.2. Characterization

The viscosity of resins for MJ printing, which utilizes a piezoelectric head, is very limited and critical. The piezoelectric printhead (Ricoh-Gen5) used in this study can only take feeds (either ink or resins) with a viscosity between 11~13 cP at the printing temperature of 70~80 °C. Thus, a Wells-Brookfield^TM^ cone/plate viscometer was employed to characterize the viscosity of the prepared resins at the printing temperature (70 °C). Three tests were conducted for each formula, and the averages and standard deviations were calculated.

The measurements of the degree of conversion (DC) were done (N = 3) using an FTIR spectrometer with a transparent accessory. The resin was coated on a KBr holder forming a thin film with a defined thickness. The wavenumber range of the spectrum was 4000~400 cm^−1^, and the FTIR spectra of the sample were recorded before and after curing (30 s curing time). The degree of conversion (DC) in our resin system can be assessed by the degree of the acrylate C=C bond dissociation, relating to the variation of the FTIR absorbance intensities of the corresponding peak (C=C, at 1638 cm^−1^). It was calculated according to the following equation:

(1) $$DC\;\left(\%\right)=\left[1-\frac{{\left(1638\;cm^{-1}/1738\;cm^{-1}\;\right)}_{Peak\;intensity\;after\;curing}}{{\left(1638\;cm^{-1}/1738\;cm^{-1}\;\right)}_{Peak\;intensity\;before\;curing}}\right]\;\times \;100\%$$

where the 1738 cm^−1^ peak represents the C=O bond as an internal standard for the quantitative analysis. The bond is contributed by the PUA, and it does not break during the photopolymerization.

The volume shrinkage was evaluated (N = 3) by the change of density of the sample during photopolymerization, based on Archimedes’ method and the ISO 3521 standard. A uniaxial tension test (N = 3) was conducted using a universal testing machine (Model Insight 10, MTS Systems Corp., Eden Prairie, MN, USA), and ASTM D638-IV dogbone shape samples were prepared by filling molds for the test. A spectrophotometer (i1 Basic Pro 2, X-rite) and X-rite’s 3-step ColorChecker® Grayscale balance card were used to quantify the color appearance of the molded samples with the dimension of 20 mm in diameter.

**Figure 1:**
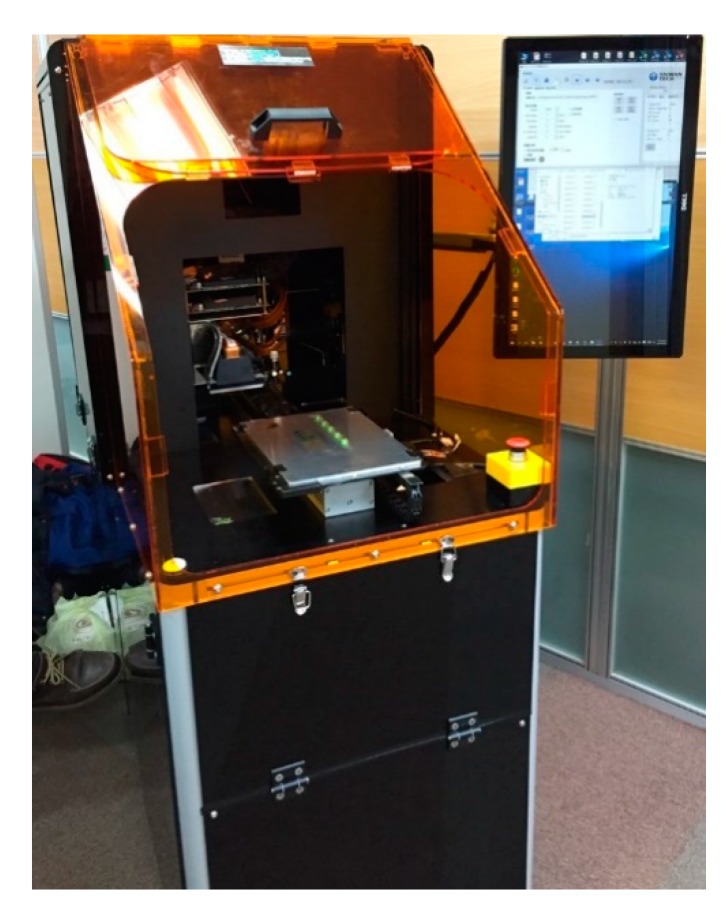
Homemade full-color material jetting (MJ) printer.

The full-color printing test for the prepared resins was performed on the homemade full-color MJ printer, which is equipped with six piezoelectric printheads (Ricoh-Gen5, 600 dpi), for the five CMYKW primary color resins and a support material. The system is shown in Figure 1. The developments of the control system of the machine were done in our previous studies, including mechanical mechanism designs, intelligent control of the mechatronics systems, automatic print head alignment algorithms, and 3D color dithering methods for improving color consistency.

## 3. Results and Discussion

### 3.1. The Effects of the Concentration of Photo-Initiator (TPO)

**Figure 2:**
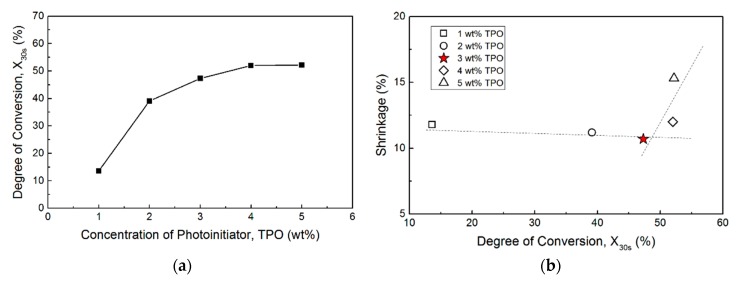
(**a**) The effect of TPO concentration on the degree of conversion, X_30s_; (**b**) a plot of rapid volume shrinkage vs. X_30s_.

A suitable concentration of the photo-initiator needed to be determined to achieve a higher curing rate but a limited volume shrinkage after curing. The properties of a photopolymer material, such as its hardness, elasticity, and chemical resistance, are mainly provided by the oligomers in the photocurable resin. In the photopolymerization process, the oligomers are typically functionalized by the acrylates at the ends of the molecular chains. In this study, acrylate-functionalized urethane oligomers, such as polyurethane acrylate (PUA), were chosen to prepare the printing resins, with the intention to benefit from the unique features of the co-existing acrylic and polyurethane groups, such as excellent abrasion resistance, toughness, and elasticity. The effects of the concentration of the photo-initiator in the resin were investigated. A resin (comprising 20 wt% aliphatic PUA, 70 wt% IBOA, and 10 wt% TPGDA) containing additionally added TPO with different weight ratios (0, 1, 2, 3, 4, and 5 wt%) was prepared. In the test, a certain amount of resin was cured under ultraviolet (UV) light for 30 s, and the DC was checked using FTIR. The results are shown in Figure 2a. The standard deviations of DC at 1, 2, 3, 4, and 5 wt% of TPO were 1.97%, 6.26%, 4.55%, 4.57%, and 1.61%, respectively. The DC increases until the concentration of the photo-initiator reaches 4 wt%. Furthermore, an excessive curing rate would cause extreme volume shrinkage of the cured resin, resulting in a lower printing accuracy. The extreme volume shrinkage could be attributed to the rapid conversion of van der Waals interactions among monomer molecules to covalent bonds instead of the uniform polymerization of all the composites. In Figure 2b, extreme volume shrinkage can be observed as the degree of conversion is higher than 50%. The 3 wt% TPO was concluded as the most adequate proportion of the photo-initiator in our resin recipes to prepare resins with a fast curing rate but moderate volume shrinkage.

### 3.2. Aliphatic PUA Oligomer vs. Aromatic PUA Oligomer

**Figure 3:**
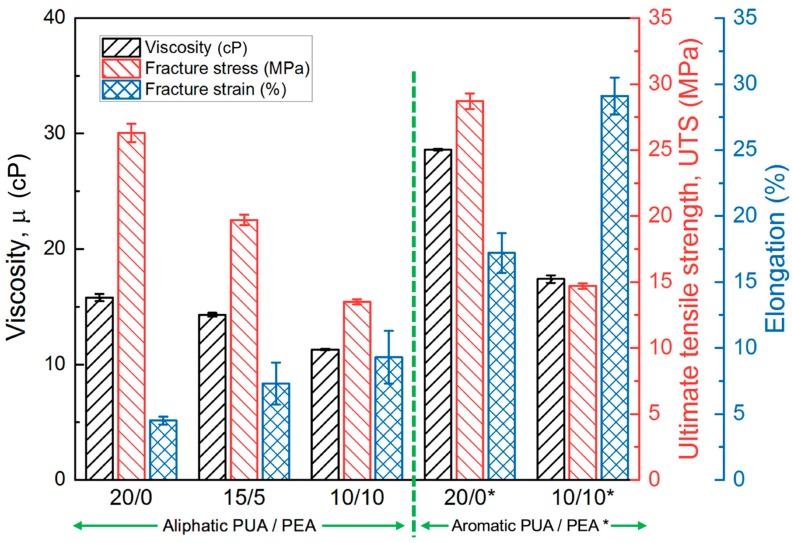
Comparisons of the physical and mechanical properties of the resins composed of different oligomers.

In the material jetting AM processes, resins should meet the viscosity requirement of the piezoelectric heads, which is 11~13 cP at the printing temperature of 70~80 °C in this study. The viscosity of the resin, comprising 20 wt% aliphatic PUA, 70 wt% IBOA, and 10 wt% crosslinker (marked as 20/0 in Figure 3), adopted in the previous section was 15.8 cP at 70 °C, slightly higher than the required viscosity. To adjust the viscosity, reactive diluents, monomers, or oligomers with a relatively low molecular weight were usually considered. In this study, tetrafunctional polyester acrylate (PEA) was introduced to partially substitute the PUA for improving the rheological property. Instead of using a monomer, oligomers with a relatively low molecular weight, like the PEA, can not only reduce the viscosity as a diluent, but also maintain the mechanical properties at their original level relatively. In Figure 3, the viscosity and mechanical properties are compared. The 5 and 10 wt% substitutions of PEA for aliphatic PUA are marked as 15/5 and 10/10, respectively, while the 20/0* and 10/10* are the original aromatic PUA-based resin and the 10 wt% substitution of PEA for aromatic PUA, respectively. The substitution of PEA reduced the viscosity of the resins effectively. A resin (10/10) with the desired viscosity of 11.3 cP could be obtained. However, the ultimate tensile strength (UTS) was reduced dramatically. The increase of the elongation by 50% could be attributed to the inherent elasticity of the PEA. As for the aliphatic PUA, the substitution of PEA revealed consistent influences on the viscosity, UTS, and elongation. The viscosity of the original aromatic PUA-based resin (20/0*) could only be decreased from 25 to 15 cP with a 10 wt% substitution of PEA, which was close but not in the range of 11~13 cP. Because the existence of a crosslinker can improve the mechanical properties, the resin 10/10 with the lowest viscosity can still possess the opportunity for further improvement. Therefore, it was selected for further study.

### 3.3. Crosslinking

**Figure 4:**
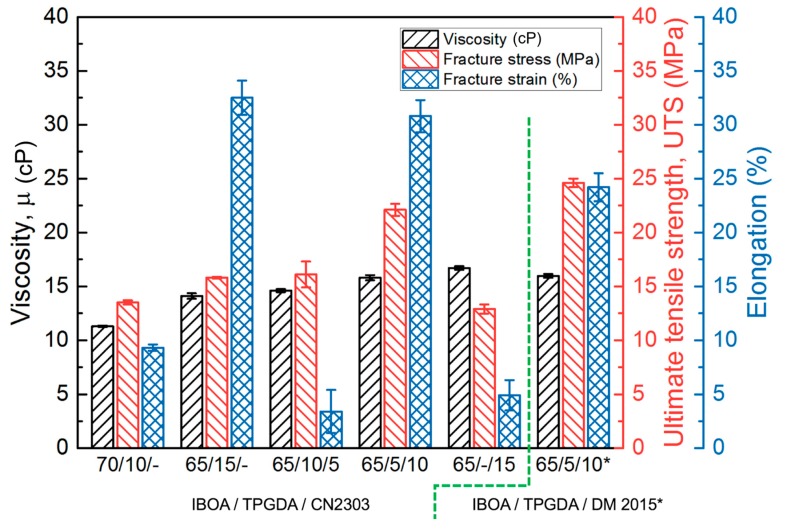
The effects of substitutions of the crosslinkers (TPGDA, CN2303, and DM 2015) for the monomer (IBOA) on the physical and mechanical properties of the prepared resin.

The bifunctional crosslinker (TPGDA) was selected as a basic crosslinker in our resin preparation. The resin 10/10 comprises 10 wt% aliphatic PUA, 10 wt% PEA, 70 wt% IBOA, and 10 wt% TPGDA, hereafter renamed 70/10/-; its mechanical properties are shown in Figure 4. To increase the crosslinking density, TPGDA was increased from 10 to 15 wt% (65/15/-) in the first trial. The results demonstrated in Figure 4 showed that the UTS improved by approximately 10% but the elongation was enhanced significantly. However, it was noticed that the viscosity of the resin also increased, which suggested that the further substitution of the TPGDA for the monomer IBOA for improving the UTS could not be allowed.

Hyperbranched crosslinker has been considered as a promising additive for effectively increasing the crosslink density of the resin during polymerization, consequently enhancing its tensile strength. In addition, the viscosity of the hyperbranched crosslinkers is usually lower due to less molecular chain entanglement, as compared with the linear crosslinkers with similar molecular weight. That suggests that the presence of hyperbranched crosslinkers would improve the crosslink density of the resin and guarantee a constant viscosity to it. Two hyperbranched crosslinkers, CN2303 and DM 2015, were selected to substitute the linear crosslinker TPGDA, with six functional sites and fifteen functional sites of each molecule, respectively. The resin 65/15/- was utilized in the study of the substitution of hyperbranched crosslinkers. The TPGDA present in the resin 65/15/- was substituted with the hexa-functional crosslinker (CN2303) at different substitution levels, 0%, 33.3%, 66.6%, and 100%, and the results are shown in Figure 4, marked as 60/15/-, 65/10/5, 65/5/10, and 65/-/15, respectively. It can be noticed that the viscosity of the resin was almost the same with the substitution of the hexa-functional crosslinker for the TPGDA, but obvious changes appeared in its mechanical properties. The results indicated the presence of a hyperbranched crosslinker due to its spherical molecular structure induced a steric effect. Although it was hard to predict how the concentration of the hyperbranched crosslinker would affect the mechanical properties of the resin, there was an optimized substitution of the hexa-functional crosslinker by 66.6% found in our experiment, namely the resin 65/5/10. It had a UTS of approximately 22 MPa and an elongation of approximately 31%. Furthermore, the 15-functional crosslinker (DM 2015) was also introduced into our study, and the optimized result was found with the substitution of the 15-functional crosslinker for the TPGDA by 66.6%, namely the resin 65/5/10*. It had a UTS of approximately 24 MPa and an elongation of approximately 24%. Both the resins 65/5/10 and 65/5/10* exhibited adequate viscosities as well as the desired mechanical properties. We decided to use the resin 65/5/10 to prepare the five primary color inks for full-color MJ printing.

### 3.4. Color Appearances of Prepared Resins

**Figure 5:**
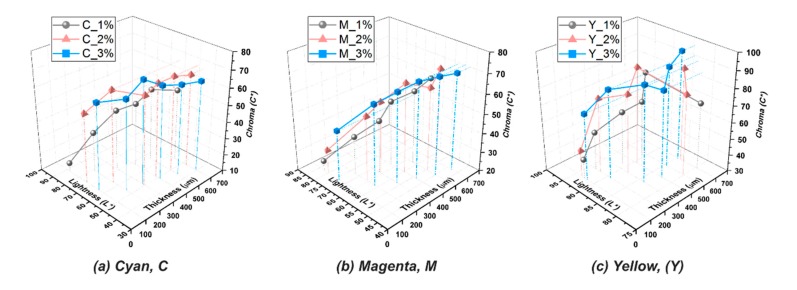
Thickness–lightness–chroma 3D plots for evaluating the color appearances of the prepared resins in (**a**) cyan, (**b**) magenta, and (**c**) yellow primary colors.

The resin 65/5/10 was selected as the base and colored with commercially available pigment inks to form the five CMYKW primary color resins for printing. To optimize the addition of the pigment inks, the effect of concentration (1, 2, and 3 wt%) of the pigment inks on the color properties of the resins was investigated. The color space of CIE L*C*h, which is calculated from CIELAB and widely used to specify colors based on three color attributes—lightness (L*), chroma (C*), and hue angle (h), was introduced for this study. These properties mainly depend on the preselected primary color pigment inks, the base color, the thickness of the resin layer, and the transparency of the resin 65/5/10. Thus, for each case (five cases in total, CMYKW), the testing samples with different thicknesses (100, 200, 300, 400, 500, and 600 μm) were prepared for characterization and the relative results were plotted into thickness–lightness–chroma 3D plots, as shown in Figure 5. The chroma is observed to be increased when the thickness of the sample is increased, at the expense of a reduction in lightness. This follows the expectations based on the subtractive color theory. For cyan resins, as indicated in Figure 5a, the resin colored with the addition of 2 wt% of the cyan pigment ink exhibits a better chroma and lightness as compared to the other two cases (1 and 3 wt%). For magenta resins, as indicated in Figure 5b, the resin colored with the addition of 3 wt% of the magenta pigment ink exhibits a better chroma and lightness as compared to the other two cases (1 and 2 wt%). A similar trend can also be found in the yellow resins, as indicated in Figure 5c. Consequently, the resins in CMY colors for the final printing test were prepared by 2 wt%, 3 wt%, and 3 wt% addition of CMY pigment inks, respectively.

**Figure 6:**
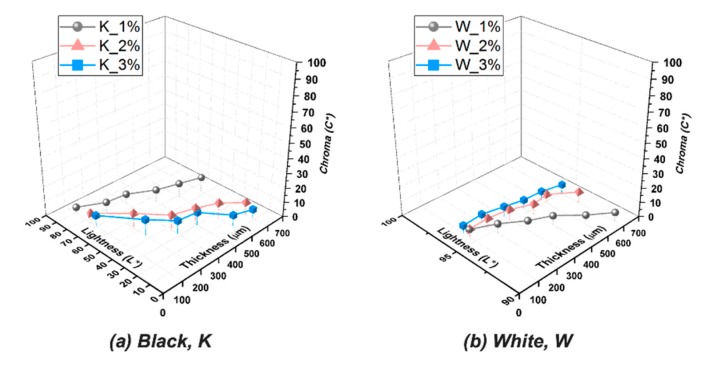
Thickness–lightness–chroma 3D plots for evaluating the color appearances of the prepared resins in (**a**) black and (**b**) white primary colors.

A broad range of lightness in the black resin is essential in the color-mixing process of a full-color printing. As indicated in Figure 6a, the resins colored with the additions of 2 and 3 wt% of black pigment ink exhibit a broader range of lightness as compared to the case of the 1 wt% addition. In more detail, it can be found that the case of the 2 wt% addition is with a smooth and gradual increment in lightness as the sample thickness increases. Thus, it was selected for the final full-color printing test. In contrast to black resins, for the white resins, an as high as possible lightness is required. As indicated in Figure 6b, the resin colored with the addition of 3 wt% of white pigment ink exhibits the highest lightness in any thickness. The resin with 3 wt% of white pigment ink was accordingly selected for the final full-color printing test.

**Figure 7:**
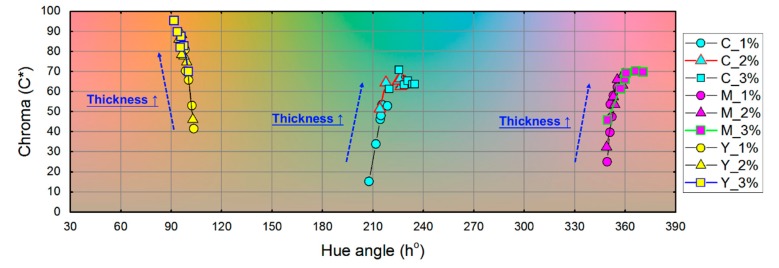
Hue angle (h)-chroma (C*) diagram of the prepared resins in CMY colors.

To evaluate the color gamut enabled by the three prepared resins in the CMY primary colors, the hue angle (h) and chroma (C*) were calculated and the results are shown in Figure 7. As discussed earlier, in our printing thickness arrangement (100~600 μm), the thicker the printing layer is, the higher chromaticity is obtained. Both cyan and yellow resins exhibit a good color appearance, but the magenta resin seems to suffer from a color shift to the red color. The color shift is the result of the yellowing caused by UV light exposure.

**Figure 8:**
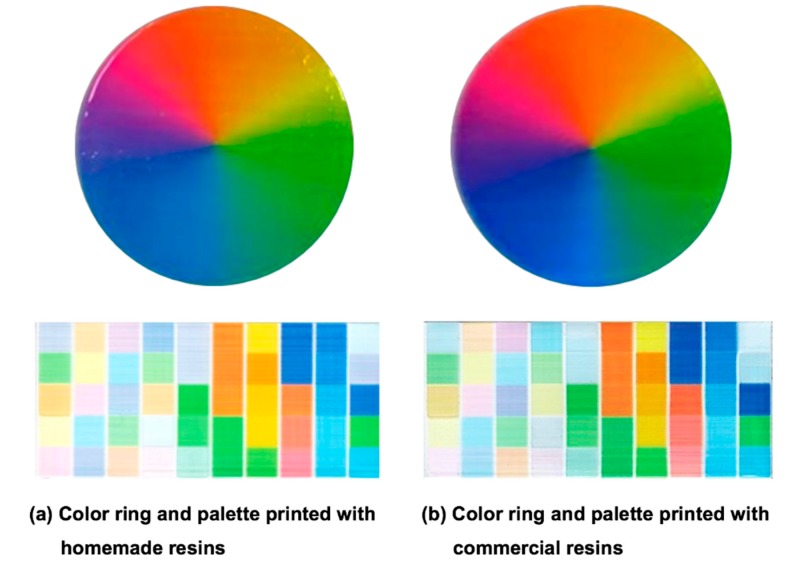
Color ring and palette printed with (**a**) homemade resins and (**b**) commercial resins.

Before employing the prepared resins for the printing test, a final check of rheological properties (viscosity and surface tension) is essential to protect the jet heads from malfunctioning. The results of the check are shown in Table 2, and the prepared resins qualified for the regulations of the jet heads, with a viscosity between 11~13 cP and a surface tension between 28~35 mN/m. For the demonstration of full-color appearance, the full-color ring and the 50-color palette were printed using the prepared resins in CMYKW primary colors. The results are shown in Figure 8a, and it demonstrates that the homemade resins enable high-quality full-color printing. For the evaluation of color developing, a clear commercial resin (VeroClear RGD810, Stratasys) was employed and colored with CMYKW colors through the same coloring process, and the standard prints are shown in Figure 8b. The appearance of the color ring printed with the commercial resins is more saturated than ours is, except for the yellow-based colors. This can be attributed to the transparency of the inherent blue color of the commercial resin and the yellowing of our resins. Thus, further improvement on the color reproduction of the resins will be done in future studies for a more reliable full-color printing.

## 4. Conclusions

Photocurable resins for MJ were successfully prepared and colored with the CMYKW colors to achieve full-color printing using a homemade MJ printer. Aliphatic-PUA was demonstrated to be a promising oligomer providing the resin system with an adequate viscosity for MJ, and the substitution of PUA with PEA proved to be an excellent reactive diluent without significantly sacrificing the mechanical properties of the printed resins. The linear crosslinker TPGDA and two hyperbranched crosslinkers improved the UTS and elongation of the printed resins. The prepared resins, named 65/5/10, with an optimized UTS (22 MPa) and elongation (31%), colored with CMYKW colors, display a satisfying full-color printing with the homemade MJ printer. This paper provides an insight into the preparation process of photocurable resins for the development of full-color material jetting.

## Figures and Tables

**Table 1:** Chemical list of this st.

Chemicals		Abbreviation	Function	Functionality	Suppliers	Trade Name
**Base**	Aliphatic polyurethane acrylate	Aliphatic-PUA	Oligomer	2	DBC	DM 553
	Aromatic polyurethane Dimethacrylate	Aromatic-PUA	Oligomer	2	DBC	DM 7201M
	Polyester acrylate	PEA	Oligomer	4	DSM-AGI Co.	AgiSyn 003
	Isobornyl Acrylate	IBOA	Monomer	1	DBC	DM IBOA
	Tripropylene glycol diacrylate	TPGDA	Crosslinker	2	DBC	DM TPGDA
	Hexafunctional Polyester acrylate	6F-PEA	Crosslinker	6	Sartomer Americas	CN2303
	Fifteen-functional Polyester acrylate	15F-PEA	Crosslinker	15	DBC	DM 2015
	Diphenyl-(2,4,6-trimethylbenzoyl)-phosphine oxide	TPO	Free radical photoinitiator		Chembridge	Cgemcure-TPO
**Additive**	Acrylic Block Copolymer	PX-4701	Dispersant		BASF	PX-4701
	Di-functional Silicone Acrylate	DM-9137	Leveling agent		DBC	DM 9137
	CMYK primary color UV ink		Pigment		ChengFeng Group	
	W primary color UV ink		Pigment		ITRI	

**Table 2:** Viscosity and surface tension of the formulated resins ready for inkjet printing.

Formulated Resins	Viscosity @ 70 °C, cP	Surface Tension, mN/m
Resin 65/5/10	15.8	29.9
Resin 65/5/10-C_2%	12.3	28.1
Resin 65/5/10-M_3%	12.0	28.1
Resin 65/5/10-Y_3%	12.1	27.7
Resin 65/5/10-K_2%	12.6	28.3
Resin 65/5/10-W_3%	11.8	27.3
